# Acute pseudogout of the foot following Parathyroidectomy: a case report

**DOI:** 10.1186/s40842-017-0048-x

**Published:** 2017-11-18

**Authors:** Sari Priesand, Jennifer Wyckoff, James Wrobel, Brian Schmidt

**Affiliations:** Department of Internal Medicine, Division of Metabolism, Endocrinology, and Diabetes, University of Michigan Hospital and Health Systems, Domino’s Farms Lobby G, Suite 1500, 24 Frank Lloyd Wright Drive, Ann Arbor, MI 48106 USA

**Keywords:** Pseudogout, Parathyroidectomy, Calcium pyrophosphate dehydrate, Diabetes mellitus

## Abstract

**Background:**

The current case report is the first in the literature to report the association between parathyroidectomy and an episode of probable pseudogout in the foot in a patient with diabetes mellitus.

**Case presentation:**

The authors present a case of pseudogout of the left foot following a subtotal parathyroidectomy in an 80-year-old female with type 2 diabetes mellitus and primary hyperparathyroidism. Pseudogout, also known as calcium pyrophosphate dehydrate deposition disease, is an unusual metabolic sequela following parathyroidectomy. Pseudogout attacks have been noted in the immediate post-operative period following parathyroidectomy, but has rarely been identified in the foot. The diagnosis was challenging and there were multiple differential diagnoses that were entertained in this case.

**Conclusions:**

This case illustrates the importance of reviewing the surgical history as there might be a link between the previous surgeries and current foot problems. This case also serves as a reminder of the importance of calcium and phosphate metabolism in podiatric health. Most specifically it demonstrates again the association of pseudogout and parathyroidectomy in a patient with diabetes mellitus. Although a rare occurrence, it is an important reminder that metabolic imbalances of calcium levels can manifest in any bone.

## Background

Calcium pyrophosphate dehydrate (CPPD) deposition disease is also known as chondrocalcinosis or pseudogout. The incidence of pseudogout in the United States is 275 cases per 100,000 people, roughly half the incidence of gout [1, 2]. Pseudogout occurs most often between the sixth and eighth decades of life and the male to female ratio is 1.5:1 [2, 3]. CPPD crystals are deposited in cartilage, synovial and capsular tissues as well as within tendons and ligaments. The most common areas of involvement are the knees, followed by the upper extremity joints and the metatarsophalangeal joint of the foot [1]. The crystals are radiopaque and appear as linear calcifications within soft tissues. Joint destruction, although uncommon, resembles osteoarthritis [1].

Although infrequent, pseudogout is a well-documented diagnostic clue of hyperparathyroidism or sequela following parathyroidectomy [4–10]. Hyperparathyroidism is known to increase the risk of CPPD, presumably due to long standing increased serum calcium [11]. Therefore, the exact mechanism by which a parathyroidectomy would precipitate acute CPPD is unclear. One commonly accepted theory is that following parathyroidectomy the relative fall in calcium levels may decrease the solubility of calcium pyrophosphate, precipitating the shedding of crystals out of the cartilage and into the synovial fluid, resulting in an inflammatory response [12]. Other possible explanations might be that the sudden decrease in the effect of parathyroid hormone (PTH) on the phosphate metabolism, either locally in cartilage or perhaps due to the drop in the renal excretion of phosphate, unbalances the equilibrium between the chondrocyte’s production of inorganic pyrophosphate (iPP) and its hydrolysis to orthophosphate by tissue non-specific alkaline phosphatase. iPP is integral to the formation of CPPD crystals and therefore a relative, even transient, increase in iPP might be expected to increase the deposition of CPPD crystals [13]. Acute synovitis develops when the crystals produce an acute inflammatory response [1].

The diagnosis is challenging, especially in post-surgical patients in whom fever, leukocytosis, and pain may be attributed to other causes [14, 15]. The differential diagnosis for this condition includes: gout [16, 17], hungry bone disease [18], Charcot neuroarthropathy, seronegative arthritis, osteoarthritis, rheumatoid arthritis, and cellulitis/septic arthritis.

Joint aspiration and microscopic identification of crystals are recommended to establish a definitive diagnosis. The microscopic demonstration of weakly positive birefringent crystals of varying shapes under polarized light (1 to 20 μm in length) is diagnostic [1]. The recommended conservative treatment for pseudogout is pharmacologic management with NSAIDs, as well as the judicious use of intra-articular steroid injections [1, 19]. Colchicine can be used [20], although it is relatively inconsistent in relieving symptoms; NSAIDs may be more helpful [1].

This case is unique as it is the first case in the literature to describe a case of pseudogout of the foot following parathyroidectomy in a patient with type 2 diabetes. Our case was complicated because our patient also had type 2 diabetes, making the diagnosis difficult. This emphasizes the importance of reviewing surgical history, as well as ruling out other possibilities in the differential diagnoses, in order to attribute the symptoms to a specific diagnosis and choose appropriate treatment.

## Case presentation

Our patient is an 80-year-old female with past medical history of well-controlled type 2 diabetes mellitus for 37 years, chronic kidney disease stage 3, monoclonal gammopathy of uncertain significance, chronic kidney disease stage 3 (stable for a decade), hypertension, obesity (BMI 39), GERD, hyperlipidemia, treated endometrial cancer, and primary hyperparathyroidism. She presented with an erythematous, painful, edematous left foot. Patient denied any history of smoking, alcohol, or illicit drug use. Patient denied any previous history of pedal complaints.

Patient had biochemical and radiographic evidence of primary hyperparathyroidism for 8 years and was being followed by both an endocrinologist and an endocrine surgeon. She had no clinical complaints related to her hypercalcemia, no history or ultrasonographic evidence for kidney stones, and excellent bone density. However, progressive hypercalcemia developed and prompted surgical intervention. Her pre-operative lab values are displayed in Table 1.

**Table 1:** Patient’s labs prior to parathyroidectomy

Lab test	Lab value
Serum Calcium (Ca) [8.6–10.3 mg/dl]	10.4–11.9 mg/dl
Parathyroid Hormone (PTH) [10-65 pg/ml]	154 pg/ml
25-hydroxyvitamin D [30–50 ng/ml]	47 ng/ml

She underwent a subtotal parathyroidectomy of grossly enlarged right superior parathyroid sand left superior parathyroid glands. A morphologically normal right inferior parathyroid gland and a mildly enlarged left inferior parathyroid gland (that appeared to be largely replaced by adipose) were left in situ. PTH levels were monitored throughout the procedure. An appropriate drop in PTH levels from 553 pg/ml (normal 12-75 pg/ml) baseline (right internal jugular) to 37 pg/ml was observed. Pathology revealed two enlarged, hyper-cellular glands. Her surgical procedure was without complication and she was discharged the day after surgery after an uncomplicated overnight stay. At discharge on post op day 1, PTH was 14 pg/dl and serum calcium was 8.3 mg/dl.

**Fig. 1:**
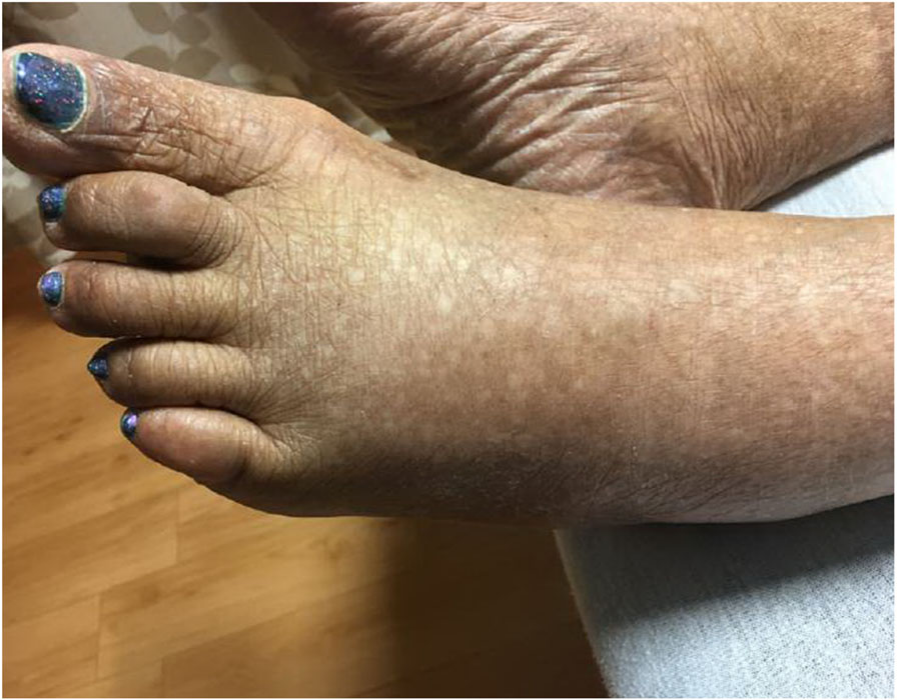
Clinical image left foot

**Fig. 2:**
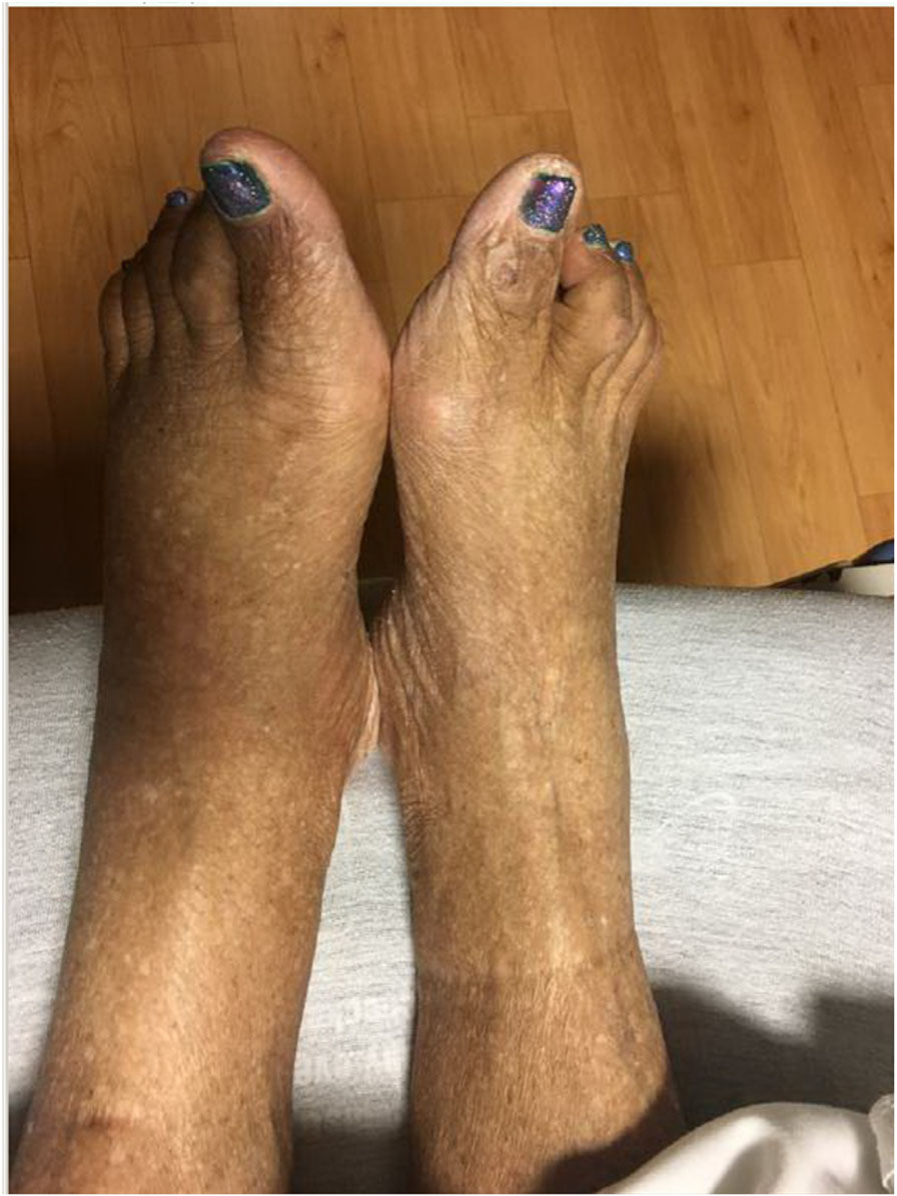
Clinical image left foot vs. right foot

Seven days post operatively, the patient developed left foot pain, swelling, warmth, and inability to bear weight (Figs. 1 and 2). She denied any precipitating trauma. She had no prior history of cellulitis or Charcot neuroarthropathy. She denied any numbness, burning, or tingling to her feet or any symptoms of peripheral neuropathy. She noted that the foot pain became progressively worse, particularly with standing. She otherwise denied any fever, chills, nausea, vomiting, chest pain, shortness of breath, abdominal pain, or diarrhea. Her initial labs on readmission are shown in Table 2.

**Table 2:** Patient’s labs upon readmission status post parathyroidectomy

Lab test	Lab value
WBC [4.0–10.0 K/uL]	11.5 K/uL
BUN [7–20 mg/dl]	25 mg/dl
Cr [0.5–1.1 mg/dl]	1.42 mg/dl
ESR [0–20 mm/h]	78 mm/h
CRP [≤3.0 mg/dl]	12.6 mg/dl
Ca [8.6–10.3 mg/dl]	7.5 mg/dl

**Fig. 3:**
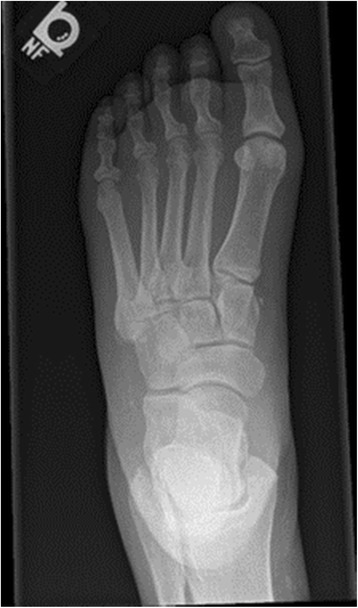
X-ray left foot

**Fig. 4:**
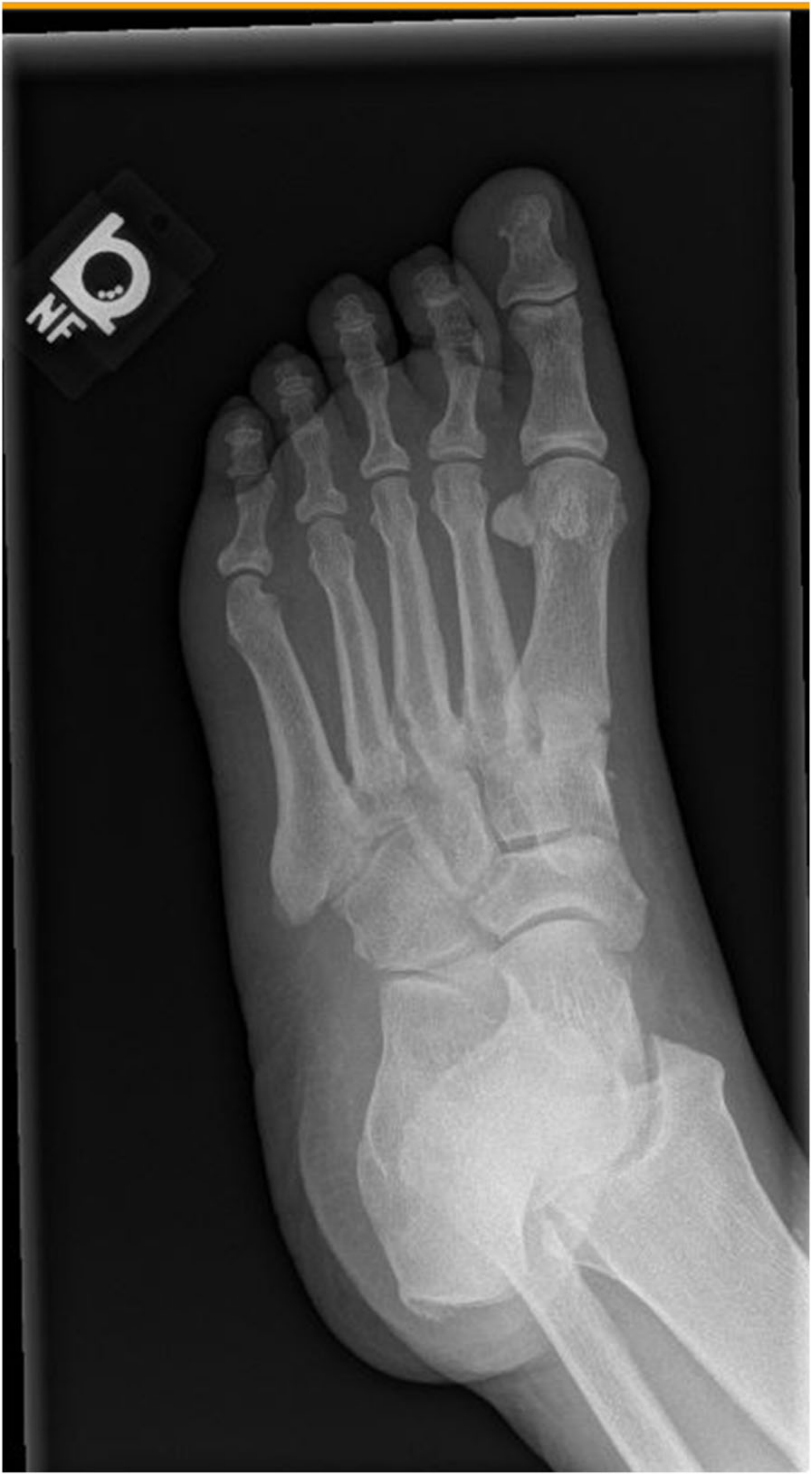
X-ray left foot

**Fig. 5:**
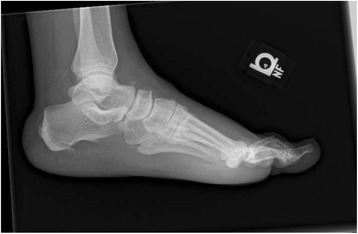
X-ray left foot

There was radiographic evidence of soft tissue swelling with no findings concerning for osteomyelitis or Charcot neuroarthropathy (Figs. 3, 4, 5). She was admitted with the presumed of cellulitis of the left foot and was started on intravenous antibiotics (Zosyn and Vancomycin).

**Fig. 6:**
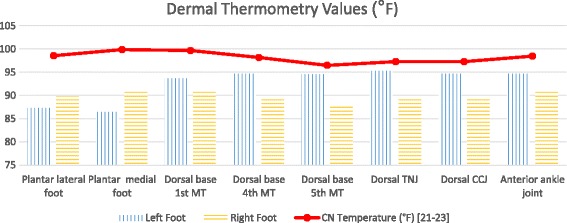
Dermal thermometry values. Abbreviations: MT metatarsal; TNJ talonavicular joint; CCJ calcaneocuboid joint; CN Charcot Neuroarthropathy. Legend: vertical stripes left foot; horizontal stripes right foot; solid line charcot neuroarthropathy temperature levels

**Fig. 7:**
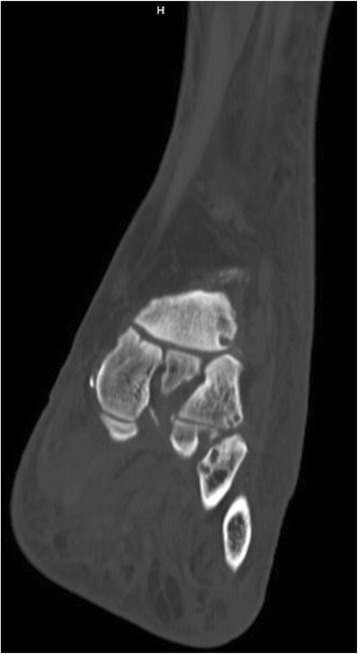
CT scan left foot

**Fig. 8:**
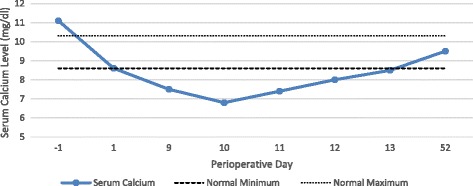
Peri-operative serum calcium levels. Legend: solid line patient values; dashed points minimum normal values; dotted points maximum normal values

Three days following her admission, a podiatry consult was placed for evaluation of the left foot. Upon physical exam patient had 2/4 dorsalis pedis and posterior tibial pedal pulses bilaterally, capillary fill time was within normal limits and the left foot was warm to touch compared to the right foot. Protective sensation was evaluated via 5.07 monofilament, which revealed intact sensation to bilateral feet. Vibratory sensation was evaluated via timed clanging tuning fork and was measured to be 19 s at the right first metatarsophalangeal joint and 20 s at the left first metatarsophalangeal joint. Thus, the vibratory sensation was within normal limits to bilateral feet. On focused evaluation of the left foot, it appeared the foot was erythematous, warm and markedly swollen. There was no lymphadenopathy noted. There were no portals of entry visualized and no open wounds identified. There was pain on palpation specifically over the dorsal midfoot laterally. There was no crepitus noted on range of motion of the midtarsal or tarsometatarsal joint and no pain on range of motion of the individual metatarsals. No crepitus or fluctuance noted.

On initial assessment, the differential diagnosis of cellulitis versus Charcot neuroarthropathy was entertained. The patient did not improve on broad spectrum antibiotics though her white blood cell count did normalize. She denied any systemic symptoms. The diagnosis of septic arthritis at this point seemed unlikely. Given lack of evidence of neuropathy, the diagnosis of Charcot neuroarthropathy seemed less likely. Dermal thermometry was performed bedside and the recordings (Fig. 6) were analyzed according to work of Armstrong et al. and Najafi et al. [21–23] The x-rays were reviewed and there were no notable findings. Patient denied a history of gouty arthritis or pedal osteoarthritis. She reported that she did not have foot pain prior to admission. On further assessment and review of recent parathyroidectomy surgical history, podiatry recommended a CT scan (Fig. 7) for evaluation of the underlying osseous structures, an endocrinology evaluation due to patient’s persistent hypocalcemia, and a rheumatology consult for evaluation for pseudogout. Patient was made non weight bearing in a CAM (controlled ankle movement) walker at this time as well.

Endocrinology was consulted for her hypocalcemia, and the recommendation for calcitriol 0.25mcg BID, continue calcium citrate-vitamin D3 supplement 2 tabs TID, and to continue vitamin D3 1000 units daily. They also recognized that hungry bone syndrome is another documented outcome following parathyroid gland removal, but deemed it unlikely in this case.

Upon evaluation by the rheumatology team, the diagnosis of a crystalline arthritis was evident. The recommendations included starting 0.6 mg BID colchicine and then to continue with 0.6 mg colchicine daily until first post discharge follow up, 10 mg daily prednisone, and 100 mg daily allopurinol after acute symptoms resolved. They recommended a uric acid level be drawn, which returned as 7.5 mg/dL (normal 2.2–6.0 mg/dL). The CT scan revealed a constellation of findings suggesting sequela of midfoot and ankle gout arthropathy. At this point the diagnosis of gout versus pseudogout was discussed and it seemed that due to patient’s recent parathyroidectomy, pseudogout was more likely. NSAIDs were avoided in this case due to patient’s compromised renal status. The rheumatology team also felt a joint aspiration at this point was not necessary, and attempting treatment with pharmacologic therapy first was most appropriate.

After beginning pharmacologic treatment as recommended by both the endocrinology and rheumatology teams, calcium levels began to slowly normalize (Fig. 8) and patient’s left foot pain began to decrease. The patient’s left foot edema reduced and the erythema resolved shortly thereafter. Patient at this point was transitioned to protected weight bearing of the left lower extremity in the CAM Walker with use of a walker and gait training with physical therapy.

## Discussion

This case highlights the clinical presentation of pseudogout following parathyroid gland removal. The diagnosis of pseudogout is difficult due to the various diagnoses that mimic its clinical presentation. Specifically, when type 2 diabetes mellitus is present the additional diagnoses of septic joint/cellulitis and acute Charcot neuroarthropathy must be ruled out. CN, when in Eichenholtz stage 0, is clinically difficult to diagnose, as there are no visible radiographic findings. Acute CN classically presents as an erythematous, edematous, warm, neuropathic/insensate foot. CN, when in stages 1–3, display various levels of radiographic joint fragmentation with subsequent remodeling and consolidation [24, 25]. Lomax et al. reviewed how the radiographic destruction seen in cases of CPPD can resemble CN [26]. In this case, the absence of peripheral neuropathy ruled out the possibility that CN was playing a role. The importance of reviewing the surgical history was vital in this case as it explained the patient’s hypocalcemia in addition to the left foot symptoms.

In this case, performing a joint aspiration of the tarsometatarsal joint would have been definitive, as microscopic evaluation of the joint crystals would have differentiated between pseudogout and gout as well as definitively rule out septic joint. However, the risk of introducing bacteria into a non-septic joint was a concern and the rheumatology recommendation for pharmacologic therapy prior to joint aspiration was appreciated in this case. Zuber’s study on knee joint aspirations and injections stated that the introduction of infection after injection is less than 1 in 10,000 procedures. He also addresses that a contraindication to aspiration is an inaccessible joint [27]. The tarsometatarsal joint anatomically is composed of multiple joint articulations including the tarsometatarsal, intertarsal, and proximal intermetatarsal joints. There are dorsal, plantar, and interosseous ligaments making the tarsometatarsal joints technically challenging to obtain an adequate synovial fluid sample [28]. Therefore, the rationale for administering pharmacologic medication prior to attempting synovial fluid analysis was deemed appropriate in this clinical setting. In terms of treatment, this patient responded quite well to colchicine and prednisone. NSAIDs in this case were avoided due to patient’s history of renal compromise, but in an otherwise healthy patient NSAIDs could have been utilized.

Although not a factor in this particular case, as there were no significant destructive osseous changes post-operatively or bone symptoms pre-operatively, we wanted to briefly comment on hungry bone syndrome as it has not yet been addressed in the podiatric literature. Hungry bone syndrome occurs following parathyroidectomy, when sometimes profound hypocalcemia occurs due to the acute decline in PTH. As Brasier described in 1988, in the hyperparathyroid individual, PTH increased bone formation and resorption with a net efflux of calcium from the bone. Sudden withdrawal of PTH, as in parathyroidectomy, causes an imbalance between osteoblast-mediated bone formation and osteoclast-mediated bone resorption, leading to a marked net increase in bone uptake of calcium, phosphate, and magnesium [18]. Clinical features of this condition include hypocalcemia, hypophosphatemia, hypomagnesemia, and hyperkalemia. In Brasier’s study, the incidence of hungry bone syndrome following parathyroidectomy was 12.6% [18].

## Conclusion

This case illustrates the importance of reviewing the surgical history as there might be a link between the previous surgeries and current foot problems. This case also serves as a reminder of the importance of calcium and phosphate metabolism in podiatric health, as pseudogout, gout, and hungry bone syndrome were all entertained in this case. The clinical presentation of CPPD can be variable and is a great mimicker of other forms of arthritis as well as Charcot neuroarthropathy. Most specifically it demonstrates again the association of pseudogout and parathyroidectomy in a patient with diabetes mellitus and it describes it for the first time in the podiatric literature. Although a rare occurrence, it is an important reminder that metabolic imbalances of calcium levels can manifest in any bone.

## Abbreviations

BUN: Blood urea nitrogen
Ca: Calcium
CAM: Controlled ankle movement
CCJ: Calcaneocuboid joint
CN: Charcot neuroarthropathy
CPPD: Calcium pyrophosphate dehydrate
Cr: Creatinine
CRP: Creactive protein
ESR: Erythrocyte sedimentation rate
IPP: Inorganic pyrophosphate
MT: Metatarsal
PTH: Parathyroid hormone
TNJ: Talonavicular joint
WBC: White blood cell
